# Magnetic Resonance Elastography of Regional Aortic Mechanics and True- and False-Lumen Stiffness in Aortic Dissection: A Feasibility Study

**DOI:** 10.64898/2026.09.09.26362667

**Published:** 2026-09-13

**Authors:** Adnan A. Hirad, Huiming Dong, Manjunathan Nanjappa, Baqir Kedwai, Kristin Thompson, Joel Kruger, Jarvy Chen, Arunark Kolipaka, Doran Mix

## Abstract

**Objective:**

Regional aortic mechanics and the mechanical environments of the true and false lumens are not represented by maximal diameter alone. We evaluated whether magnetic resonance elastography (MRE) could provide paired descending thoracic and abdominal aorta stiffness estimates and separate true- and false-lumen estimates in thoracic aortic dissection.

**Methods:**

We acquired 60-Hz descending thoracic and abdominal aortic MRE data in 10 healthy subjects and in 2 patients with chronic type B aortic dissections. Three motion-encoding directions and four temporal offsets were reduced to first-harmonic complex wave fields. After directional filtering, we traced a curved centerline through the aortic mask. Local spatial frequency was estimated along arc length and converted to effective shear stiffness. The primary regional outcome was the mean effective stiffness over the central 20%-90% of the path from the quality-ranked slice in each region. In dissection, the whole-aorta mask and flap were defined on a registered sagittal scout; a vascular surgeon assigned the true and false lumens, and separate, manually centered lumen paths followed one union-mask phase correction and unwrapping step. We compared regional values with paired tests.

**Results:**

All 10 regional datasets and all 56 available slices were processed without failure. The 10 healthy volunteers (mean age, 26.6 ± 4.6 years; 6 men) had descending thoracic stiffness of 15.43 ± 3.30 kPa and abdominal stiffness of 19.20 ± 3.14 kPa. The mean thoracic-minus-abdominal difference was -3.77 ± 3.40 kPa (95% confidence interval, -6.20 to -1.34 kPa; paired t-test, P=.007; exact Wilcoxon signed-rank test, P=.010). Eight participants had a higher abdominal value, and two had a higher thoracic value. In Subject A with a chronic aortic dissection, slice-averaged true- and false-lumen stiffness was 21.62 and 19.07 kPa, respectively (false-minus-true, -2.54 kPa; absolute percentage difference, 12.5%). In Subject B with a chronic aortic dissection, corresponding values were 12.17 and 17.40 kPa (false-minus-true, +5.22 kPa; 35.3%).

**Conclusions:**

We demonstrated the feasibility of MRE to provide paired regional analysis of the aorta in healthy people and to separate stiffness profiles of the true and false lumens in Type B thoracic dissection cases. Abdominal effective stiffness exceeded descending thoracic stiffness in the volunteer cohort. Opposite true- and false-lumen contrast in the two subjects with chronic Tye B aortic dissections demonstrates measurement feasibility but does not establish a consistent compartment pattern, disease threshold, or intrinsic wall material property.

**Clinical Relevance:**

Surveillance of aortic disease is primarily anatomic, although expansion and rupture are mechanical processes. A regional MRE measurement may complement anatomic analyses by localizing wave-propagation behavior to a specific aortic segment or dissection lumen. The present study establishes the feasibility of noninvasive measurement of stiffness in a true and false lumen of dissected aorta.

**Highlights:**

- Local-frequency magnetic resonance elastography (MRE) yielded paired abdominal and descending thoracic effective-stiffness estimates in all 10 volunteers.
- Abdominal effective stiffness exceeded descending thoracic effective stiffness in this young feasibility cohort.
- True and false-lumen stiffness contrast was measurable in both dissection cases, but its direction differed between cases.
- The results show the potential of MRE to monitor aortic diseases such as dissection-associated aneurysms and abdominal and thoracic aneurysms.

## Full Text Availability

The license terms selected by the author(s) for this preprint version do not permit archiving in PMC. The full text is available from the preprint server.

